# Platelet hyperreactivity and risk of ischemic placental disease: A prospective cohort study

**DOI:** 10.1002/pmf2.70255

**Published:** 2026-02-13

**Authors:** Christina A. Penfield, Andre A. Robinson, Ariel Schaap, Elliot Luttrell‐Williams, Anais Hausvater, Yuhe Xia, Matthew Muller, Madison S. House, Valeryia Avtushka, Lila Murphy, Clarice G. Zhou, Justin S. Brandt, Gwendolyn P. Quinn, Ashley S. Roman, Dana R. Gossett, Jeffrey S. Berger

**Affiliations:** ^1^ Division of Maternal‐Fetal Medicine, Department of Obstetrics and Gynecology NYU Grossman School of Medicine New York New York USA; ^2^ Department of Obstetrics and Gynecology NYU Langone Health New York New York USA; ^3^ Division of Hematology, Department of Medicine NYU Grossman School of Medicine New York New York USA; ^4^ Leon H. Charney Division of Cardiology, Department of Medicine NYU Grossman School of Medicine New York New York USA; ^5^ Sarah Ross Soter Center for Women's Cardiovascular Research NYU Grossman School of Medicine New York New York USA; ^6^ Division of Biostatistics NYU Grossman School of Medicine New York New York USA; ^7^ Department of Obstetrics and Gynecology NYU Grossman School of Medicine New York New York USA; ^8^ Center for the Prevention of Cardiovascular Disease NYU Grossman School of Medicine New York New York USA

**Keywords:** aggregation, hyperreactivity, hypertensive disorders of pregnancy, ischemic placental disease, platelets, preeclampsia

## Abstract

**Introduction:**

Platelet hyperreactivity is linked to inflammation and cardiovascular risk in nonpregnant populations, but its relationship to placentally mediated pregnancy outcomes is undefined. We prospectively evaluated platelet hyperreactivity in pregnancy and subsequent ischemic placental disease (IPD), and examined aspirin's (ASA) effect on platelet activity by baseline platelet phenotype.

**Methods:**

Pregnancy Outcomes and Platelet PhenotYpes (POPPY) was a prospective cohort study with blood collection in the first and second trimesters. Platelet function was assessed via light transmission aggregometry (LTA) and platelet hyperreactivity was defined a priori as >60% aggregation in response to 0.4 µM epinephrine. The primary outcome was a composite IPD endpoint (hypertensive disorders of pregnancy [HDP], placental abruption, small‐for‐gestational‐age [SGA] birth, and stillbirth). We used multivariable logistic regression to estimate the association between baseline platelet hyperreactivity and IPD. In addition, first‐trimester platelet transcriptome (pre‐ASA) was compared between participants with and without platelet hyperreactivity. In a high‐risk subset defined per US Preventative Task Force criteria and recommended ASA 81 mg daily, we evaluated ASA's effects on serum thromboxane (TxB2) and platelet aggregation by hyperreactivity status.

**Results:**

Of 66 pregnant participants recruited, 61 had first‐trimester LTA; 20 (32.8%) had platelet hyperreactivity. Participants with hyperreactivity were more likely to develop IPD than those without (55% vs. 24%; adjusted odds ratio [aOR], 3.77; 95% confidence interval [CI], 1.05–13.53), with consistent directionality across individual components despite low event frequencies. Participants with platelet hyperreactivity showed greater platelet aggregation to adenosine diphosphate (ADP), collagen, and low‐dose arachidonic acid (AA). Platelet transcriptomic profiling distinguished participants with versus without platelet hyperreactivity and revealed differential expression of pathways related to platelet activity, energy metabolism, and immune regulation. Among 45 high‐risk participants recommended ASA, those with hyperreactivity exhibited higher AA‐induced aggregation (24% vs. 12.5% platelet aggregation, *p* = 0.01) despite similar serum TxB2 levels.

**Conclusion:**

First‐trimester platelet hyperreactivity was present in approximately one third of the participants and was independently associated with increased risk of IPD. Participants with platelet hyperreactivity demonstrated distinct transcriptomic signatures and greater platelet aggregation despite ASA use. Together, these findings support a contributory role for platelets in placental ischemic pathology and highlight the need to elucidate mechanisms and develop platelet‐targeted preventive strategies.

**Trial Registration:**

ClinicalTrials.gov identifier: NCT06194643.

## 1 INTRODUCTION

Ischemic placental disease (IPD) encompasses a constellation of pregnancy complications that include new‐onset hypertensive disorders of pregnancy (HDP), placental abruption, and birth of small‐for‐gestational‐age (SGA) neonates [1, 2]. In the United States, IPD affects approximately 12%–15% of pregnancies and is associated with an increased risk of preterm birth as well as substantial maternal and neonatal morbidity and mortality [3]. These complications are unified by shared pathophysiologic pathways involving uteroplacental ischemia and placental insufficiency [1, 2, 4]. Platelets are increasingly recognized as key mediators in the development of IPD outcomes, primarily through the formation of microthrombi that impair placental trophoblast invasion and contribute to placental infarcts that result in the hypoxia and ischemia characteristic of IPD [5]. Aspirin (ASA) reduces the risk of preeclampsia and SGA birth [5], along with other significant pregnancy complications such as preterm birth and stillbirth [6], yet the mechanisms through which ASA modifies platelet activity in the placental environment are poorly understood.

ASA exerts its antiplatelet effects primarily through irreversible inhibition of cyclooxygenase‐1 (COX‐1), thereby blocking the conversion of arachidonic acid (AA) to prostaglandin H2 (PGH2), leading to decreased production of prostaglandins, including thromboxane (TxB2), a key mediator of platelet activation and aggregation [7]. However, platelet activation is heterogeneous, and studies from nonpregnant populations have recognized that a subset of individuals exhibit a hyperreactive platelet phenotype characterized by high platelet aggregation even in response to low‐dose agonist stimulation [8, 9]. Platelet hyperreactivity is a reproducible and generalizable phenotype that reflects increased responsiveness across multiple platelet activation pathways, is associated with a distinct genetic signature, and is predictive of future cardiovascular events [9]. Because IPD is driven in part by platelet‐mediated microvascular dysfunction [10], it is plausible that this same hyperreactive phenotype may be clinically relevant during pregnancy. We hypothesized that platelet hyperreactivity during pregnancy confers an increased risk of IPD and may attenuate the preventive efficacy of ASA. To test this, we prospectively evaluated the association between first‐trimester platelet hyperreactivity and subsequent development of IPD and examined the effect of ASA on platelet aggregation among participants with and without baseline platelet hyperreactivity.

## 2 MATERIALS AND METHODS

The Pregnancy Outcomes and Platelet PhenotYpes (POPPY) study was a prospective cohort study evaluating platelet phenotype during pregnancy (Figure S1). From January 24, 2023 to June 3, 2024, individuals with viable singleton pregnancies and not already on ASA were enrolled at prenatal visits. Participants were excluded if they had contraindications to ASA, anemia (hemoglobin <10 g/dL), thrombocytopenia (<100 × 10^9^/L) or thrombocytosis (>500 × 10^9^/L), inability to consent in English, or planned delivery at an outside hospital. As part of clinical care, participants were classified as either “high risk” (recommended ASA prophylaxis) or “low risk” (not recommended ASA) according to US Preventative Task Force (USPTF) criteria [6]. Those identified as high risk were recommended to initiate low‐dose ASA (81 mg daily) between 12 and 16 weeks’ gestation [6].

Baseline clinical and demographic information was collected through clinician chart review at the time of enrollment. To evaluate potential disparities, participants self‐reported racial and/or ethnic group, which was reported in a single combined racial/ethnic category aligned with current national reporting guidelines [11]. The institutional review board approved the study and participants provided written informed consent.

### 2.1 Study procedures

Blood samples were collected in the first trimester, targeting 11–14 weeks (before ASA initiation), and again in the second trimester, targeting 24–28 weeks, when high‐risk participants were recommended to take ASA 81 mg daily. Before each blood draw, participants were assessed for conditions that might affect platelet function, including recent antibiotic use or COVID‐19 infection. If such factors were present, blood collection was postponed. All blood samples were delivered to the research lab within 30 min of collection for processing.

#### 2.1.1 Platelet analysis

Complete blood count (CBC) parameters were analyzed on a Sysmex XN‐1000 hematology analyzer. We assessed platelet indices including count, size (measured by mean platelet volume [MPV]), and maturity (measured by immature platelet fraction [IPF] percent [%] and absolute number). Platelet function was assessed via light transmission aggregometry (LTA) as described in the Supporting Information Methods) [12, 13]. Platelet hyperreactivity in the baseline sample was defined a priori as >60% aggregation in response to 0.4 µM epinephrine [8, 14, 15].

#### 2.1.2 Definition of pregnancy outcomes

The primary clinical outcome was a composite of IPD, which includes the standard outcomes of HDP, birth of SGA neonate, and placental abruption [1]. Stillbirth (fetal demise after 20 weeks’ gestation) was incorporated into the composite because it is an outcome for which ASA prophylaxis has demonstrated benefit [6]. HDP included gestational hypertension and preeclampsia (including superimposed preeclampsia) with or without severe features, as well as eclampsia and Hemolysis, Elevated Liver enzymes, Low Platelets (HELLP) syndrome [16]. SGA birth is defined as birthweight below the 10th percentile according to the INTERGROWTH‐21st nomogram [17]. Placental abruption is a clinical diagnosis defined as premature separation of the placenta from its uterine attachment before the delivery of a fetus, often in the context of painful vaginal bleeding. Secondary endpoints included each component of the composite outcome as well as IPD with preterm preeclampsia only (excluding term deliveries and other HDP) to specifically evaluate an outcome for which ASA prophylaxis has demonstrated benefit [6]. Preterm preeclampsia was defined as preeclampsia or superimposed preeclampsia, with or without severe features, resulting in delivery before 37 weeks. We also evaluated gestational diabetes (according to the Carpenter–Coustan Criteria) [18]. All diagnoses were reviewed through 6 weeks postpartum and adjudicated by clinicians through chart review while blinded to platelet hyperreactivity status.

#### 2.1.3 ASA subset analysis

In a subset analysis, we examined a subgroup of the POPPY cohort classified as high risk for preeclampsia according to USPTF criteria who were recommended ASA 81 mg. For this subset, we evaluated the platelet activity at baseline (in the first trimester prior to ASA use) and during the second trimester targeted for 24–28 weeks [13]. To evaluate potential noncompliance, participants were asked about ASA use and we evaluated serum TxB2 and platelet aggregation measured by LTA in response to high‐dose AA 1600 µM, a widely used pharmacodynamic marker of ASA‐mediated platelet inhibition [19].

#### 2.1.4 Platelet transcriptome analysis

Platelet transcriptome analysis was performed on participants during the first trimester, prior to ASA use, and compared the results of patients with versus without platelet hyperreactivity, as described in the Supporting Information Methods.

#### 2.1.5 Statistical analysis

Data were analyzed using standard descriptive and multivariable methods. Platelet aggregation measures are reported as medians with interquartile ranges (IQRs) and were compared using the Mann–Whitney *U* test. Categorical variables are presented as counts and percentages and were compared using the chi‐square test or Fisher's exact test, as appropriate. Statistical testing was not performed for outcomes with very low cell counts. Univariate and multivariable logistic regression analyses were conducted to estimate the association between platelet hyperreactivity and the likelihood of IPD. Adjustment was performed to address key baseline factors known a priori to influence platelet parameters (age [20, 21], race/ethnicity [22], and body mass index [BMI] [23, 24]). Sensitivity analysis evaluating potential ASA non‐compliance in the high‐risk group on ASA was completed. All *p* values were two‐sided. Analyses were performed using R version 4.3.0.

## 3 RESULTS

**FIGURE 1:**
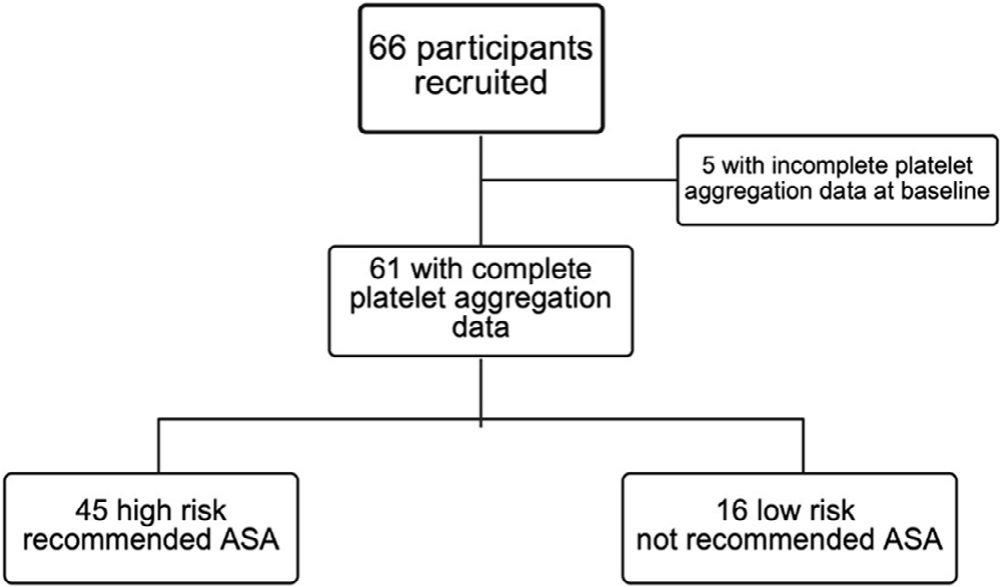
Participant flow diagram. ASA, aspirin.

Of the 66 participants enrolled in POPPY, 61 had platelet aggregation data at baseline (Figure 1). Overall, the median age was 35 years (ranging from 26 to 45 years) with diverse representation across race/ethnicities (Table 1). Using USPTF criteria, 45 participants were designated high risk and 16 were designated low risk.

**TABLE 1:** POPPY participant demographics.

	Overall	No platelet hyperreactivity *N* = 41	Platelet hyperreactivity *N* = 20
	*N* = 61		
Maternal age (median [IQR])	35.4 [32.7–38.4]	35.3 [32.5, 37.6]	35.9 [33.0, 38.9]
Self‐described race and/or ethnicity			
American Indian or Alaska Native	1 (1.6)	0 (0.0)	1 (5.0)
Asian	7 (11.5)	5 (12.2)	2 (10.0)
Black or African American	7 (11.5)	4 (9.8)	3 (15.0)
Hispanic/Latino	17 (27.9)	10 (24.4)	7 (35.0)
Middle Eastern or North African	1 (1.6)	0 (0.0)	1 (5.0)
Prefer not to answer	1 (1.6)	0 (0.0)	1 (5.0)
White	27 (44.3)	21 (51.2)	6 (30.0)
Nulliparous	37 (60.7)	25 (61.0)	12 (60.0)
Body mass index (BMI, kg/m^2^; median [IQR])	25.7 [22.0, 30.5]	25.1 [22.1, 30.2]	26.8 [20.6, 31.7]
Obese	18 (29.5)	12 (29.3)	6 (30.0)
Chronic hypertension	9 (14.8)	6 (14.6)	3 (15.0)
Pregestational diabetes	3 (4.9)	2 (4.9)	1 (5.0)
History of preeclampsia	4 (6.6)	3 (7.3)	1 (5.0)

*Note*: All data are *N*(%) unless otherwise specified.

Abbreviations: IQR, interquartile range; POPPY, Pregnancy Outcomes and Platelet PhenotYpes.

**FIGURE 2:**
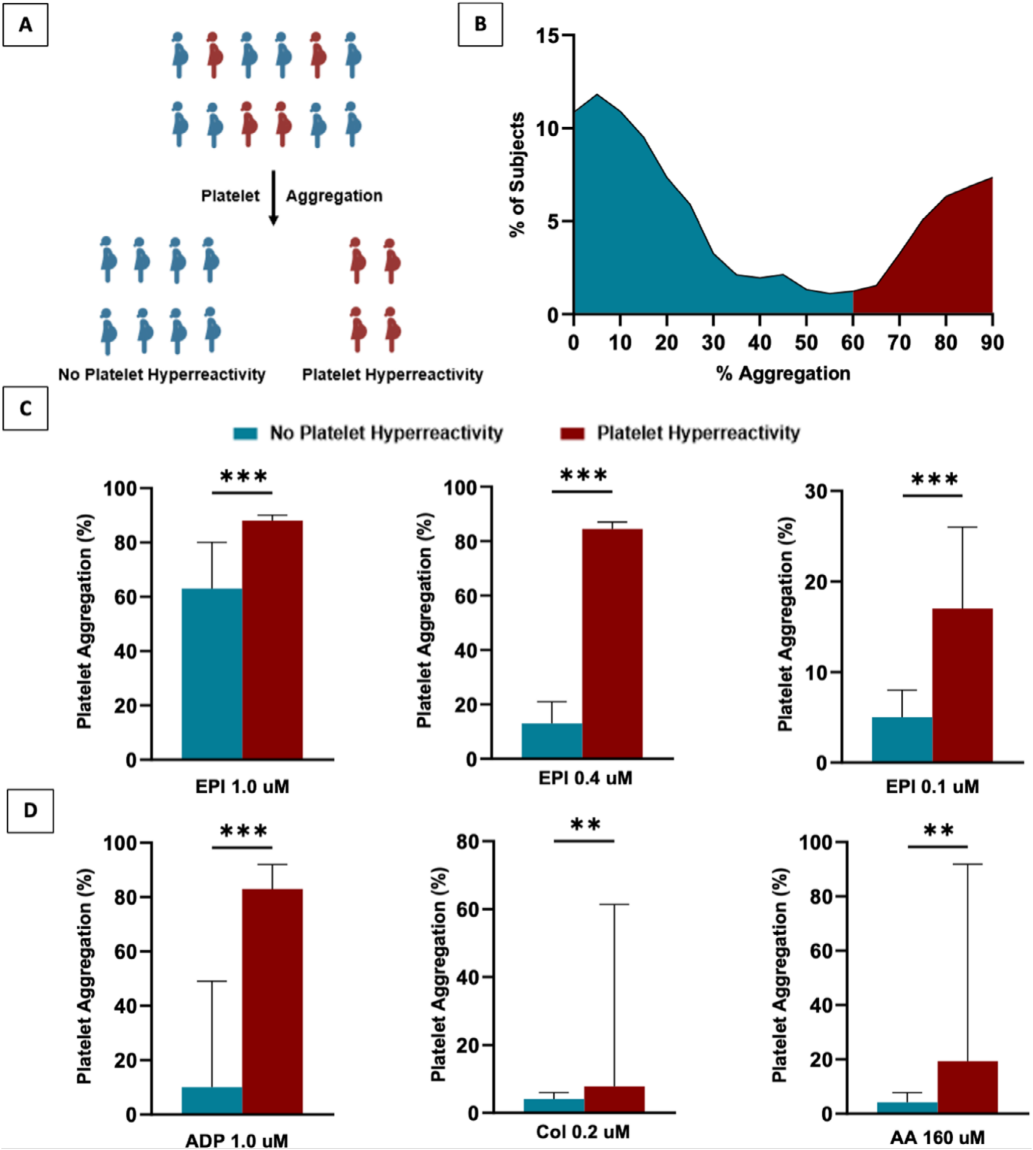
Distribution of platelet aggregation among participants. (A) Participants were stratified at baseline based on platelet hyperreactivity status (platelet aggregation >60% in response to 0.4 µM epinephrine (EPI). (B) There was a bimodal response in distribution, with a distinct minority of participants exhibiting platelet hyperreactivity and the remainder exhibiting lower platelet aggregation. (C) Platelet aggregation in response to different doses of EPI. (D) Platelet aggregation in response to 1.0 µM adenosine diphosphate (ADP), 160 µM arachidonic acid (AA), and 0.2 µM collagen (Col). **p < 0.01; ***p < 0.001.

At the first‐trimester baseline blood draw, median platelet aggregation in response to submaximal epinephrine (0.4 µM) at 300 s was 21.0 (IQR 10.0–75.0). The distribution was bimodal, with a distinct minority of pregnant participants (*n* = 20, 32.8%, Figure 2A,B) exhibiting platelet hyperreactivity (> 60% aggregation) and the remainder exhibiting lower aggregation responses. Demographic and clinical characteristics were similarly distributed between those with or without platelet hyperreactivity (Table 1).

Participants with platelet hyperreactivity also exhibited greater platelet aggregation across a range of epinephrine concentrations (Figure 2C) and in response to other agonists at submaximal doses (Figure 2D and Table 2). In contrast, other platelet parameters, including platelet count, MPV, and measures of platelet maturity (IPF percent and absolute), did not differ between participants with and without platelet hyperreactivity (Table 2).

**TABLE 2:** Platelet parameters and aggregation by platelet hyperreactivity at baseline.

Parameters	No platelet hyperreactivity (*n* = 41)	Platelet hyperreactivity (*n* = 20)	*p* value
Platelet count	242 [192–290]	251 [195–314]	0.43
Mean platelet volume	10.2 [9.9–11.3]	10.3 [9.9–11.0]	0.98
Immature platelet fraction (%)	3.0 [1.9–5.4]	3.1 [2.4–4.1]	0.58
Immature platelet fraction (absolute)	6.95 [4.8–11.2]	7.7 [6.8–10.4]	0.42
Agonist			
Spontaneous^*^	4.9 [3.0–6.8]	6.0 [5.4–7.3]	0.178
ADP (0.2 µM)^*^	4.3 [3.0–6.4]	5.6 [4.1–6.5]	0.168
ADP (1.0 µM)	10.0 [4.0–49.0]	83.0 [50.5–92.0]	<0.001
ADP (2.0 µM)	84.0 [75.0–90.0]	92.0 [91.0–93.0]	<0.001
AA (160 µM)^*^	4.2 [1.4–7.8]	19.4 [7.3–91.9]	0.001
AA (1600 µM)	89.0 [86.0–90.0]	90.0 [88.0–91.3]	0.274
AA (1600 µM) + ASA	17.0 [12.5–19.0]	21.5 [18.3–26.0]	0.007
Collagen (0.2 µM)^*^	4.1 [2.2–6.0]	7.8 [4.7–61.4]	0.004
Collagen (1.0 µM)	84.0 [78.0–89.0]	89.0 [84.5–90.0]	0.009
Epinephrine (0.1 µM)	5.0 [3.0–8.0]	17.0 [9.3–26.0]	<0.001
Epinephrine (0.4 µM)	13.0 [6.0–21.0]	84.5 [75.0–87.0]	<0.001
Epinephrine (1.0 µM)	63.0 [17.0–80.0]	88.0 [84.8–90.0]	<0.001

*Note*: Data are median [IQR]. All values measured at 300s unless otherwise specified by * which indicates maximum aggregation. Maximum aggregation is used when median aggregation is zero at 300s.

Abbreviations: AA, arachidonic acid; Abs, absolute; ADP, adenosine diphosphate; IQR, interquartile range.

We found that platelet hyperreactivity was associated with a higher incidence of the IPD composite outcome (55% vs. 24% in those without platelet hyperreactivity, *p* = 0.04; Table 3). After adjusting for age, race/ethnicity, and BMI, platelet hyperreactivity was associated with more than a threefold increased odds of developing IPD composite compared with participants without platelet hyperreactivity (adjusted odds ratio [aOR], 3.77; 95% confidence interval [CI], 1.05–13.53). Participants with platelet hyperreactivity were also more likely to develop individual components of the IPD outcome, though there were fewer individuals with each of these complications.

**TABLE 3:** Clinical outcomes according to platelet hyperreactivity status.

Outcome	No platelet hyperreactivity (*n* = 41)	Platelet hyperreactivity (*n* = 20)	Unadjusted OR (95% CI)	Adjusted OR^a^ (95% CI)
Ischemic placental disease	10 (24.4)	11 (55.0)	3.79 (1.22‐11.77)	3.77 (1.05–13.53)
HDP	9 (22.0)	9 (45.0)	2.91 [0.92, 9.19]	2.50 [0.68, 9.17]
Placental abruption	0	1 (5.0)	–	–
Stillbirth	0	1 (5.0)	–	–
Small for gestational age	2 (5.0)	3 (15.0)	–	–
Secondary outcomes				
Ischemic placental disease with preterm preeclampsia^b^	2 (5.0)	6 (30.0)	–	–
Preterm preeclampsia	1 (2.4)	4 (20.0)	–	–
GDM	3 (7.3)	3 (15.0)	–	–

*Note*: Data are *n*(%) unless otherwise specified.–, no statistical comparison was performed due to low cell counts.

Abbreviations: CI, confidence interval; GDM, gestational diabetes mellitus; HDP, hypertensive disorder of pregnancy; OR, odds ratio.

^a^ Adjusted for age, race/ethnicity, and body mass index.

^b^ Must have diagnosis of preeclampsia (or superimposed preeclampsia) with or without severe features with preterm birth <37 weeks, placenta abruption, stillbirth, or small for gestational age.

### 3.1 ASA subgroup

There were 45 participants classified as high risk by USPTF criteria and thus recommended ASA for the prevention of preeclampsia. Consistent with the overall cohort, 13 of these 45 high‐risk participants (28.9%) had first‐trimester platelet hyperreactivity, measured prior to ASA use. Participants with platelet hyperreactivity also had higher baseline platelet aggregation in response to other submaximal agonists, including adenosine diphosphate (ADP), collagen, and AA (Table S1).

Prior to ASA initiation, serum TxB2 levels and platelet aggregation in response to high‐dose AA did not differ between participants with and without platelet hyperreactivity (Table 4). Following ASA use, both serum TxB2 and high‐dose AA‐induced platelet aggregation decreased in each group, demonstrating ASA's inhibitory effects on platelet activity. While serum TxB2 levels were similar between groups after ASA use (0.3 ng/mL for both groups, *p* = 0.23), those with platelet hyperreactivity demonstrated greater high‐dose AA‐induced platelet aggregation after ASA use (24% vs. 12.5%, *p* = 0.007) and an attenuated difference in platelet inhibition (74% decrease vs. 85% decrease, *p* = 0.012). Participants with hyperreactivity also had greater aggregation in response to other agonists following ASA use (Table S2). A sensitivity analysis excluding the three participants with persistently high AA‐induced aggregation, suggestive of nonadherence, yielded similar results.

**TABLE 4:** Thromboxane (TXB_2_) production and platelet aggregation with arachidonic acid (AA) among high‐risk participants recommended aspirin by platelet hyperreactivity status at baseline.

	Visit	No platelet hyperreactivity (*N* = 32)	Platelet hyperreactivity (*N* = 13)	*p* value
TXB_2_ production	Baseline	34.4 [21.3, 50.2]	22.6 [19.3, 28.0]	0.359
	Follow‐up	0.3 [0.1, 0.6]	0.3 [0.2, 1.6]	0.234
	Relative difference between baseline and follow‐up	−0.99 [−1.00, −0.98]	−0.99 [−0.99, −0.97]	0.082
Platelet aggregation (%) with AA (1600 µM)	Baseline	90.0 [85.8, 90.3]	89.0 [88.0, 90.0]	0.8
	Follow‐up	12.5 [10.3, 20.5]	24.0 [15.0, 52.0]	0.007
	Relative difference between baseline and follow‐up	−0.85 [−0.88, −0.78]	−0.74 [−0.83, −0.42]	0.012

Finally, platelet hyperreactivity provided additional risk stratification within both the USPTF low‐ and high‐risk groups. In the high‐risk group, the incidence of IPD composite was higher among those with platelet hyperreactivity (8/13; 61.5%) than among those without platelet hyperreactivity (10/32; 31.2%). In the low‐risk group, all observed IPD events occurred among participants with platelet hyperreactivity at baseline.

### 3.2 Platelet hyperreactivity and the platelet transcriptome

To investigate the biological basis of our findings, we analyzed the platelet transcriptome in participants with platelet RNA collected and sequenced (*N* = 49) and compared the sequencing data in 16 participants with versus 33 participants without platelet hyperreactivity. Overall, 126 genes were differentially expressed between groups using a false discovery rate of 0.1 (Figure S2A,B). Participants with platelet hyperreactivity had upregulation of pathways fundamental to protein synthesis and energy generation, and downregulation of pathways related to immune response and cellular communication. Platelet Reactivity ExpreSsion Signature (PRESS) genes, previously demonstrated to be predictive of platelet hyperreactivity and cardiovascular risk [13], were significantly enriched in the platelet hyperreactivity group (Figure S2C,D).

## 4 DISCUSSION

In this study, we prospectively evaluated the association between first‐trimester platelet hyperreactivity and a composite of pregnancy complications related to IPD. We identified a distinct subset of pregnant participants who exhibited platelet hyperreactivity, characterized by heightened aggregation in response to lower dose stimulation with epinephrine. Those with platelet hyperreactivity were more than three times as likely to develop IPD as those without platelet hyperreactivity, suggesting that early platelet function phenotyping may help identify individuals at elevated risk for placental ischemic complications. Among high‐risk pregnant participants for whom ASA was recommended, those with platelet hyperreactivity demonstrated less platelet inhibition in the second trimester compared with those without platelet hyperreactivity, despite similar levels of serum TxB2 levels. These findings suggest that platelet hyperreactivity in early pregnancy reflects a biologically distinct phenotype associated with both increased IPD risk and reduced platelet inhibition by ASA.

Beyond their established roles in thrombosis and hemostasis, platelets are increasingly recognized as mediators of inflammation and immunity. Increased platelet activity has been observed across cardiovascular, rheumatologic, and infectious diseases. Trip et al. first demonstrated that spontaneous platelet aggregation measured 3 months after myocardial infarction, in the absence of antiplatelet therapy, was associated with greater cardiovascular morbidity and all‐cause mortality [25]. Subsequent studies, including work from our group, have shown that platelet aggregation testing can identify individuals with a hyperreactive platelet phenotype [8, 9, 14, 15]. Platelet hyperreactivity to submaximal epinephrine is reproducible, generalizable across platelet activation pathways, and linked to increased cardiovascular risk [8, 9, 14, 15]. In pregnancy, increasing evidence suggesting a platelet‐mediated role in IPD outcomes such as HDP has been primarily through platelet indices and activation markers. Increased platelet size (higher MPV) and immaturity (higher IPF) have been associated with HDP, even after adjustment for risk factors [26]. A meta‐analysis identified MPV as a promising biomarker for detecting and monitoring preeclampsia [27]. However, studies examining other measures of platelet function have been limited, with most assessments occurring late in pregnancy, leaving early pregnancy platelet dynamics largely unexplored. In this prospective cohort study, we extend prior evidence linking platelet function to pregnancy outcomes and demonstrate that platelet hyperreactivity measured in the first trimester is strongly associated with IPD, supporting a potential role for platelets in disease pathogenesis. Pregnant patients who demonstrated platelet hyperreactivity to epinephrine also had higher platelet aggregation in response to submaximal doses of ADP, collagen, and AA, reinforcing the generalizability of this measurement and supporting its potential role in IPD pathogenesis.

Furthermore, among pregnant patients recommended ASA during pregnancy, those with platelet hyperreactivity demonstrated reduced platelet inhibition with ASA 81 mg daily, suggesting that this standard ASA dose may be insufficient in high‐risk populations for preventing ischemic pregnancy outcomes. Pharmacokinetic data on higher‐dose ASA dosing in pregnancy have been evaluated in a prior study, which demonstrated a total drug metabolite concentration of ASA in pregnancy was reduced with 100 mg versus 150 mg [10]. In addition, several meta‐analyses and observational studies have investigated higher doses of ASA clinically. Many of these studies suggested that higher doses of ASA may be more effective than lower doses (<100 mg/day) for preventing preeclampsia, although findings have not been uniform [8, 11, 12, 13, 14]. The landmark ASPRE (Aspirin for Evidence‐Based Preeclampsia Prevention) trial used a 150 mg daily dose and demonstrated a significant reduction in preterm preeclampsia compared with placebo [15]. A subsequent US trial comparing 81 mg and 162 mg ASA strategies did not demonstrate a difference in preeclampsia rates, which may reflect heterogeneity in underlying risk profiles and biologic response to ASA within the study population [16]. Our findings support the possibility that a more nuanced, precision‐based approach to ASA dosing may be warranted, in which individual platelet phenotypes, such as platelet hyperreactivity, are considered when determining the optimal ASA dose rather than applying a uniform dosing strategy [17, 18]. Future studies are needed to investigate this possibility.

Platelet transcriptomic profiling distinguished pregnant individuals with platelet hyperreactivity, revealing differential expression of pathways involved in protein synthesis, energy production, and immune regulation. These findings highlight underlying molecular mechanisms of platelet hyperreactivity and may inform improved diagnostics and understanding of pregnancy‐related ischemic complications. Previously, our group developed the PRESS, a genetic platelet signature that distinguished platelet hyperreactivity and cardiovascular risk [9]. Consistent with prior findings, PRESS pathways were among the top candidate pathways enriched in pregnant patients with platelet hyperreactivity. Future studies should leverage this genetic signature of platelet hyperreactivity to investigate platelet‐mediated diseases and guide personalized approaches for improving pregnancy outcomes.

This study has several strengths, which include the prospective design, comprehensive assessment of platelet activity, standardized assessment of clinical outcomes, and representation of a diverse cohort of both high and low‐risk pregnant patients. Consistent with clinical practice, ASA was recommended in accordance with current USPTF guidelines, making our findings applicable to obstetric practice in the United States. Additionally, we evaluated an expanded composite of placental‐mediated outcomes with potential implications from platelet hyperreactivity beyond HDP. We also included participants who were on ASA to further explore the relationship between platelet hyperreactivity and ASA platelet inhibition in pregnant participants receiving ASA.

There were also several important limitations to consider. This study is observational and therefore cannot draw definitive conclusions about causation. The relatively small size of our sample and the number of participants with the primary outcome limit the precision of our effect estimates. We were unable to adequately evaluate important secondary clinical outcomes or compare demographics between those with and without platelet hyperreactivity given the size of the cohort. In addition, residual confounding and confounding by indication cannot be excluded, as participants with platelet hyperreactivity may have differed in unmeasured ways, including underlying inflammatory or vascular characteristics, that influenced both platelet function and risk of placental ischemic outcomes. We were also unable to make meaningful comparisons with the control group given its small size. Our study was not designed to evaluate clinical outcomes in the ASA subgroup nor whether ASA can mediate the impact of platelet hyperreactivity, which is an important next step to consider. As such, the predictive performance of platelet reactivity measures, including their positive and negative predictive values, warrants further investigation in larger, adequately powered cohorts. All pregnant patients receiving ASA took 81 mg daily, and thus, we are unable to assess different dosing strategies and hyperreactivity. Future studies should investigate the optimal dosing in pregnant patients with platelet hyperreactivity.

## 5 CONCLUSION

In conclusion, approximately one in three pregnant participants exhibited first‐trimester platelet hyperreactivity, which was associated with greater platelet aggregation and an increased risk of ischemic placental outcomes. Among those with platelet hyperreactivity, daily low‐dose ASA (81 mg) appeared less effective at inhibiting platelet aggregation, supporting a contributory role for platelets in the pathogenesis of placental ischemic complications. Future studies should focus on identifying and characterizing platelet hyperreactivity early in pregnancy and determining whether alternative ASA dosing strategies can achieve more complete platelet inhibition in individuals at risk for ischemic placental outcomes.

## CONFLICT OF INTEREST STATEMENT

The authors declare no conflicts of interest.

## ETHICS STATEMENT

The NYU institutional review board approved the study (i22‐01439) and participants provided written informed consent.

## Supporting information



Supporting information

## Data Availability

The data generated during and/or analyzed during the current study will be made available from the corresponding author on reasonable request.
