# Supplementary material for: Platelet hyperreactivity and risk of ischemic placental disease: A prospective cohort study
Source: Pregnancy (Hoboken). 2026 Feb 13;2(2):e70255. doi: 10.1002/pmf2.70255 (PMC13186447; doi:10.1002/pmf2.70255)
Supplement: Supplementary file 1 [file PMF2-2-e70255-s001.docx]

**Supplemental Appendix**

**Supplementary Methods**

- **Platelet hyperreactivity**
- **Transcriptomics**

**Supplemental Figures**

- **Supplementary Figure S1:** Study design
- **Supplementary Figure S2:** Platelet transcriptome profiling in those with platelet hyperreactivity versus those without platelet hyperreactivity

**Supplemental Tables**

- **Supplemental Table S1.** Platelet aggregation at baseline in high-risk participants
- **Supplemental Table S2.** Platelet aggregation at second trimester follow-up in high-risk participants recommended aspirin

**Supplementary Appendix References**

Supplementary Methods

*Platelet Hyperreactivity*

Platelet aggregometry was conducted on a Helena (Beaumont, TX) AggRAM light transmission aggregometer in response to variable agonist and dose stimulation, including epinephrine (0.1, 0.4, 2 µM), adenosine diphosphate (ADP; 0.1, 0.4, 2 µM), collagen (0.2, 1 µg/mL), arachidonic acid (AA; 160, 1600 [with and without ASA in vitro] µM), or saline (spontaneous platelet aggregation). Percent aggregation at 5 minutes and maximum percent of aggregation was recorded. In general, low dose agonists evoke minimal platelet aggregation, whereas higher doses agonist elicit higher levels of aggregation.

*Transcriptomics*

We analyzed the platelet transcriptome in participants during the first trimester, prior to ASA use, and compared the results of patients with versus without platelet hyperreactivity. FASTQ files from RNA-sequencing were processed using the Seq-N-Slide pipeline.^1^ Reads were trimmed using trimmomatic v0.36, aligned to the hg38 genome using STAR v2.7.3, and quantified using featureCounts v1.6.3.^2–4^ Read quality was assessed using FASTQC v0.11.7 and fastqscreen v0.16.^5^ One sample was omitted due to low quality of data and aberrant expression patterns. Samples had an average read depth 46,219,523. Gene transcripts were filtered below a determined background level to identify expressed genes. We determined a cut of 4 log2 normalized expression in at least half of samples resulting in 7,878 genes for downstream analysis.

All downstream analysis was performed in R v4.4.2 (R Foundation for Statistical Computing, Vienna, Austria). Differential expression analysis was performed via DESeq2 v1.46.0 using platelet hyperreactivity adjusted for age to determine genes of interest associated with platelet hyperreactivity. Multiple hypothesis correction was done using the Benjamini–Hochberg method. Gene Set Enrichment Analysis (GSEA) was performed on all genes using clusterProfiler v4.14.4,^6^ enriching for genesets provided via msigdbr v10.0.225.^7^ Custom GSEA were also performed on the Platelet Reactivity ExpreSsion Signature (PRESS) geneset^8^ and the platelet thromboinflammation signature.^9^ The analysis was adjusted for age and race/ethnicity. Heatmaps were created using ComplexHeatmap v2.22.0 and all plotting was done using ggplot2 v3.5.1.^10^

Supplementary Figures:

**Supplementary Figure S1: Study design**

**Supplementary Figure S2: Platelet transcriptome profiling in those with platelet hyperreactivity versus those without platelet hyperreactivity.** (A) Volcano plot of differential gene expression showing genes with nominal p < 0.05 (orange) and padj < 0.01 (red). (B) Heatmap of differentially expressed genes (padj < 0.1) with hierarchical clustering, demonstrating transcriptional separation by platelet hyperreactivity status. (C) Gene set enrichment analysis (GSEA) showing top enriched pathways. (D) GSEA walk plots for Platelet Reactivity ExpreSsion Signature (PRESS) UP (NES = 1.88, padj = 2×10⁻⁵) and PRESS DOWN (NES = -2.45, padj = 4×10⁻¹⁰) gene sets in those with platelet hyperreactivity versus no platelet hyperreactivity.

**Supplemental Table S1.** Platelet aggregation at baseline in high-risk participants

| **Parameters** | **No Platelet Hyperreactivity (n=32)** | **Platelet Hyperreactivity (n=13)** | ***P*-value** |
| --- | --- | --- | --- |
| Platelet count | 243 [188, 292] | 300 [243, 324] | 0.081 |
| Mean platelet volume | 10.5 [9.9, 11.4] | 10.2 [9.8, 10.9] | 0.427 |
| Immature platelet fraction % | 3.3 [2.1, 5.9] | 2.6 [2.3, 3.6] | 0.635 |
| Immature platelet fraction Abs | 8.4 [5.5, 12.2] | 8.0 [6.9, 10.4] | 0.818 |
| **Agonist** | | | |
| Spontaneous* | 4.8 [3.0, 6.7] | 6.3 [5.6, 8.5] | 0.043 |
| ADP (0.2µM)*** | 4.7 [3.0, 6.4] | 6.1 [5.5, 20.9] | 0.087 |
| ADP (1.0µM) | 8.0 [3.3, 42.3] | 83.0 [46.0, 92.0] | <0.001 |
| ADP (2.0µM) | 84.0 [57.8, 90.3] | 92.0 [91.0, 94.0] | 0.003 |
| AA (160µM)* | 4.7 [1.4, 7.9] | 91.1 [8.4, 93.6] | 0.001 |
| AA (1600µM) | 90.0 [85.8, 90.3] | 89.0 [88.0, 90.0] | 0.80 |
| AA (1600µM) + ASA | 17.0 [12.0, 18.5] | 22.0 [17.0, 27.5] | 0.056 |
| Collagen (0.2µM)* | 4.2 [2.7, 6.4] | 8.8 [5.0, 86.5] | 0.006 |
| Collagen (1.0µM) | 84.0 [77.3, 89.0] | 89.0 [84.0, 90.0] | 0.017 |
| Epinephrine (0.1µM) | 5.0 [2.5, 6.5] | 22.5 [15.0, 35.0] | <0.001 |
| Epinephrine (0.4µM) | 13.0 [7.5, 20.3] | 86.0 [80.0, 87.0] | <0.001 |
| Epinephrine (1.0µM) | 64.0 [18.5, 80.0] | 90.0 [84.0, 90.0] | <0.001 |
| Abs, absolute; AA, arachadonic acid; ADP, adenosine diphosphate Data are median [IQR]  All values measured at 300s unless otherwise specified by * which indicates maximum aggregation. | | | |

**Supplemental Table S2.** Platelet aggregation at second trimester follow-up in high-risk participants recommended aspirin

| **Parameters** | **No Platelet Hyperreactivity (n=32)** | **Platelet Hyperreactivity (n=13)** | **P-value** |
| --- | --- | --- | --- |
| Platelet count | 239 [205, 273] | 275 [263, 303] | 0.083 |
| Mean platelet volume | 10.4 [9.8, 11.4] | 10.3 [10.0, 10.6] | 0.545 |
| Immature platelet fraction % | 4.1 [2.1, 6.6] | 2.95 [2.3, 3.6] | 0.487 |
| Immature platelet fraction Abs | 7.9 [5.7, 13.1] | 7.5 [6.5, 8.8] | 0.971 |
| **Agonist** | | | |
| Spontaneous* | 8.0 [6.0, 11.7] | 11.2 [6.1, 13.7] | 0.45 |
| ADP (0.2µM) | 2.0 [0.0, 6.0] | 5.5 [4.0, 10.3] | 0.045 |
| ADP (0.2µM)*** | 5.7 [4.1, 9.6] | 9.1 [7.6, 18.4] | 0.045 |
| ADP (1.0µM) | 17.0 [9.5, 26.0] | 48.0 [14.5, 59.3] | 0.066 |
| ADP (2.0µM) | 43.5 [27.3, 55.0] | 69.0 [58.0, 70.1] | 0.007 |
| AA (160µM) | 4.0 [2.0, 6.0] | 4.5 [2.8, 7.8] | 0.297 |
| AA (160µM) * | 7.5 [5.0, 9.1] | 9.1 [7.7, 13.1] | 0.04 |
| AA (1600µM) | 12.5 [10.3, 20.5] | 24.0 [15.0, 52.0] | 0.007 |
| AA (1600µM) + ASA | 19.8 [15.3, 26.8] | 29.0 [26.0, 36.0] | 0.003 |
| Collagen (0.2µM) | 2.0 [0.0, 5.0] | 4.5 [0.8, 8.0] | 0.307 |
| Collagen (0.2µM) * | 5.8 [4.9, 7.5] | 10.6 [6.7, 13.4] | 0.023 |
| Collagen (1.0µM) | 8.5 [5.3, 11.5] | 24.5 [8.5, 46.3] | 0.035 |
| Epinephrine (0.1µM) | 8.0 [4.0, 10.8] | 10.0 [10.0, 19.0] | 0.01 |
| Epinephrine (0.4µM) | 13.0 [8.5, 21.0] | 18.5 [12.0, 33.0] | 0.071 |
| Epinephrine (1.0µM) | 25.0 [16.5, 31.5] | 40.0 [30.0, 51.0] | 0.006 |
| Abs, absolute; AA, arachadonic acid; ADP, adenosine diphosphate; ASA, aspirin Data are median [IQR]  All values measured at 300s unless otherwise specified by * which indicates maximum aggregation. | | | |

**Supplementary Appendix References**

1. Dolgalev, Igor. “Seq-n-slide”. Zenodo, August 14, 2024. <https://doi.org/10.5281/zenodo.13323414>.

2. Liao Y, Smyth GK, Shi W. featureCounts: an efficient general purpose program for assigning sequence reads to genomic features. *Bioinform*. 2014;30(7):923-930. doi:10.1093/bioinformatics/btt656

3. Dobin A, Davis CA, Schlesinger F, et al. STAR: ultrafast universal RNA-seq aligner. *Bioinform*. 2012;29(1):15-21. doi:10.1093/bioinformatics/bts635

4. Bolger AM, Lohse M, Usadel B. Trimmomatic: a flexible trimmer for Illumina sequence data. *Bioinform*. 2014;30(15):2114-2120. doi:10.1093/bioinformatics/btu170

5. Wingett SW, Andrews S. FastQ Screen: A tool for multi-genome mapping and quality control. *F1000R*. 2018;7:1338. doi:10.12688/f1000research.15931.2

6. Yu G, Wang LG, Han Y, He QY. clusterProfiler: an R Package for Comparing Biological Themes Among Gene Clusters. *OMICS: A J Integr Biol*. 2012;16(5):284-287. doi:10.1089/omi.2011.0118

7. Igor, Stevie Pederson, and Vince. “Igordot/msigdbr: Msigdbr 10.0.2”. Zenodo, April 15, 2025. <https://doi.org/10.5281/zenodo.15220161>.

8. Berger JS, Cornwell MG, Xia Y, et al. A Platelet Reactivity ExpreSsion Score derived from patients with peripheral artery disease predicts cardiovascular risk. *Nat Commun*. 2024;15(1):6902. doi:10.1038/s41467-024-50994-7

9. Beitzen-Heineke A, Muller M, Xia Y, et al. A platelet transcriptomic signature of thromboinflammation predicts cardiovascular risk. *JCI Insight*. Dec 22;10(24):e195824. doi: 10.1172/jci.insight.195824

10. Gu Z, Eils R, Schlesner M. Complex heatmaps reveal patterns and correlations in multidimensional genomic data. *Bioinform*. 2016;32(18):2847-2849. doi:10.1093/bioinformatics/btw313
